# Artificial Intelligence of Object Detection in Skeletal Scintigraphy for Automatic Detection and Annotation of Bone Metastases

**DOI:** 10.3390/diagnostics13040685

**Published:** 2023-02-12

**Authors:** Chiung-Wei Liao, Te-Chun Hsieh, Yung-Chi Lai, Yu-Ju Hsu, Zong-Kai Hsu, Pak-Ki Chan, Chia-Hung Kao

**Affiliations:** 1 Department of Nuclear Medicine and PET Center, China Medical University Hospital, Taichung 404327, Taiwan; 2 Department of Biomedical Imaging and Radiological Science, China Medical University, Taichung 404327, Taiwan; 3 Department of Nuclear Medicine, Feng Yuan Hospital, Ministry of Health and Welfare, Taichung 420550, Taiwan; 4 Artificial Intelligence Center, China Medical University Hospital, Taichung 404327, Taiwan; 5 Graduate Institute of Biomedical Sciences, School of Medicine, College of Medicine, China Medical University, Taichung 404327, Taiwan; 6 Department of Bioinformatics and Medical Engineering, Asia University, Taichung 413305, Taiwan

**Keywords:** faster R-CNN, Detectron2, object detection, feature pyramid network, bone scan

## Abstract

Background: When cancer has metastasized to bone, doctors must identify the site of the metastases for treatment. In radiation therapy, damage to healthy areas or missing areas requiring treatment should be avoided. Therefore, it is necessary to locate the precise bone metastasis area. The bone scan is a commonly applied diagnostic tool for this purpose. However, its accuracy is limited by the nonspecific character of radiopharmaceutical accumulation. The study evaluated object detection techniques to improve the efficacy of bone metastases detection on bone scans. Methods: We retrospectively examined the data of 920 patients, aged 23 to 95 years, who underwent bone scans between May 2009 and December 2019. The bone scan images were examined using an object detection algorithm. Results: After reviewing the image reports written by physicians, nursing staff members annotated the bone metastasis sites as ground truths for training. Each set of bone scans contained anterior and posterior images with resolutions of 1024 × 256 pixels. The optimal dice similarity coefficient (DSC) in our study was 0.6640, which differs by 0.04 relative to the optimal DSC of different physicians (0.7040). Conclusions: Object detection can help physicians to efficiently notice bone metastases, decrease physician workload, and improve patient care.

## 1. Introduction

The advancement of medical technology has led to the development of successful treatments for numerous diseases. However, treatments with a complete cure rate are lacking for some conditions, and, among these, the mortality rate is the highest in cancer. As the severity of cancer increases, it typically metastasizes to various organs and bones, resulting in poor treatment outcomes and prognosis. Among routine medical imaging modalities, such as magnetic resonance imaging (MRI), positron emission tomography/computed tomography (PET/CT), and bone scan, bone scans are the most commonly used because of cost and time considerations [1,2]. Bone scans are conducted using ^99m^Tc-labeled bisphosphonates (e.g., methylene diphosphonate [MDP]), which can accumulate in areas of active bone formation or repair. Bone scans involve planar scans only. Therefore, unanticipated small lesions that appear in three-dimensional imaging may be overlooked in two-dimensional scans with overlapping bone structures. The efficacy of bone scan for bone metastasis detection is compromised by its poor specificity [3,4,5]. The simultaneous annotation/localization of lesions to be treated with localized treatment (e.g., radiotherapy, surgery and etc.) without the need for additional translation/communication from the routine written report, assisting radiologists to directly annotate lesions for treatment doctors, and reducing omission of lesion detection owing to fatigue of human experts.

## 2. Materials and Methods

### 2.1. Methods Overview

With the advancement of object detection technology, more researchers are using object detection technology for nuclear medical imaging. For example, Yuan et al. used YOLOv2 to detect skull fractures [6]. Rani et al. used region-based convolutional neural network (R-CNN), fast region-based convolutional neural network (Fast R-CNN), faster region-based convolutional neural network with region proposal network (Faster R-CNN), Faster R-CNN with region proposal network (RPN), YOLO, single-shot multibox detector, and efficient-det variety of object detection models to detect the localization of stroke lesions on MRI [7]. Bin Liu et al. performed automatic quantification of knee osteoarthritis severity using improved Faster R-CNN [8].

In 2015, Redmon et al. developed a new technology, namely YOLO: unified, real-time object detection, for simple and fast object detection [9]. In this method, object detection is considered a regression task for segmenting bounding boxes from space, and class probabilities are computed. Critical difficulties remain in the field of object detection. However, the potential outcomes for future use of object detection are considerable. The YOLO series is now widely used. Its main advantage is its relative speed, but its accuracy for small object detection is slightly lower to that of other methods. This study presents comparisons with Ren et al.’s Faster-RCNN feature pyramid network (FPN) [10], Liu et al.’s Swin Transformer of Faster R-CNN [11], and Wang et al.’s YOLOR [12]. Using Detectron 2 [13] provided by Facebook AI Research, this study performed Faster R-CNN, as proposed by R. Girshick [14], based on R50 (backbone with ResNet50) FPN. Detectron2 is powered by the PyTorch deep learning framework, including state-of-the-art object detection algorithms, such as Mask R-CNN, Faster R-CNN, Fast R-CNN, DensePose, RetinaNet, and PointRend.

### 2.2. Experimental Data

**Figure 1:**
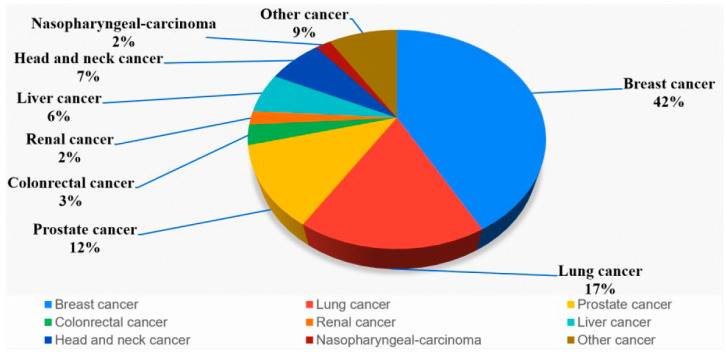
The distribution of cancer in all patients.

This study retrospectively collected the data of 920 patients who underwent bone scans at China Medical University Hospital between 2009 and 2019. Only those who have cancer were included, and those who do not have cancer were excluded. The gold standard comes from formal case report interpretation, including clinical data such as tumor markers, PET/CT images, MRI images, cancer types and biopsy. Routine whole-body scans were performed 2–4 h after the intravenous administration of 20 mCi of ^99m^Tc Tc-labeled MDP with a scan speed of 14–17 cm/min on either a Millennium MG, Infinia Hawkeye 4, or Discovery NM/CT 670 Pro scanner (GE Healthcare, Chicago, IL, USA). Each patient’s bone scan produced two images, namely anterior and posterior images, with a resolution of 1024 × 256 pixels, and a total of 1840 images were obtained. Of the collected images, 870 (450 set bone metastases and 420 set no bone metastases) were used for training, and 50 (20 set bone metastases and 30 set no bone metastases) were used for testing. We used Digital Imaging and Communications in Medicine raw values as the model input instead of converting the data into other image formats. We used SimpleITK, a Python 3.7.0 based package, to read the pixel values of the images in the Digital Imaging and Communications in Medicine file. The image dimensions were 1024 × 256 pixels, and images had both front and back sides. The same image was overlapped three times, generating images with the dimensions of 1024 × 256 × 3. Subsequently, we used the natural logarithm to standardize the overall image. The predominant cancer type (see Figure 1) was breast cancer (42%), followed by lung cancer (17%), prostate cancer (12%), head and neck cancer (7%), liver cancer (6%), colorectal cancer (3%), nasopharyngeal carcinoma (2%), renal cancer (2%), and other cancers (9%). The nursing staff members labeled the images after they had interpreted the contents of the doctor’s report. The detection results consisted of five classes, namely bone metastasis, bladder, kidney, residual radiopharmaceutical after injection, and foreign object.

SimpleITK was used for image loading and processing, Matplotlib was used for graph visualization, and Numpy was used for all mathematics and array operations. Python was used as the programming language, and Detectron2 was used for programming the models. The execution hardware was an Nvidia V100 graphics processing unit. In the model, we set 32 sliced images as the input batch size, and the overall training process ran 26,999 iterations.

During model training, random data augmentation included rotation and brightness adjustment. Since bone metastasis detection involves small object detection, we adjusted the size of the anchor box to smaller pixel sizes, which were 8, 10, 16, 32, and 64 pixels.

### 2.3. Experimental Design

**Figure 2:**
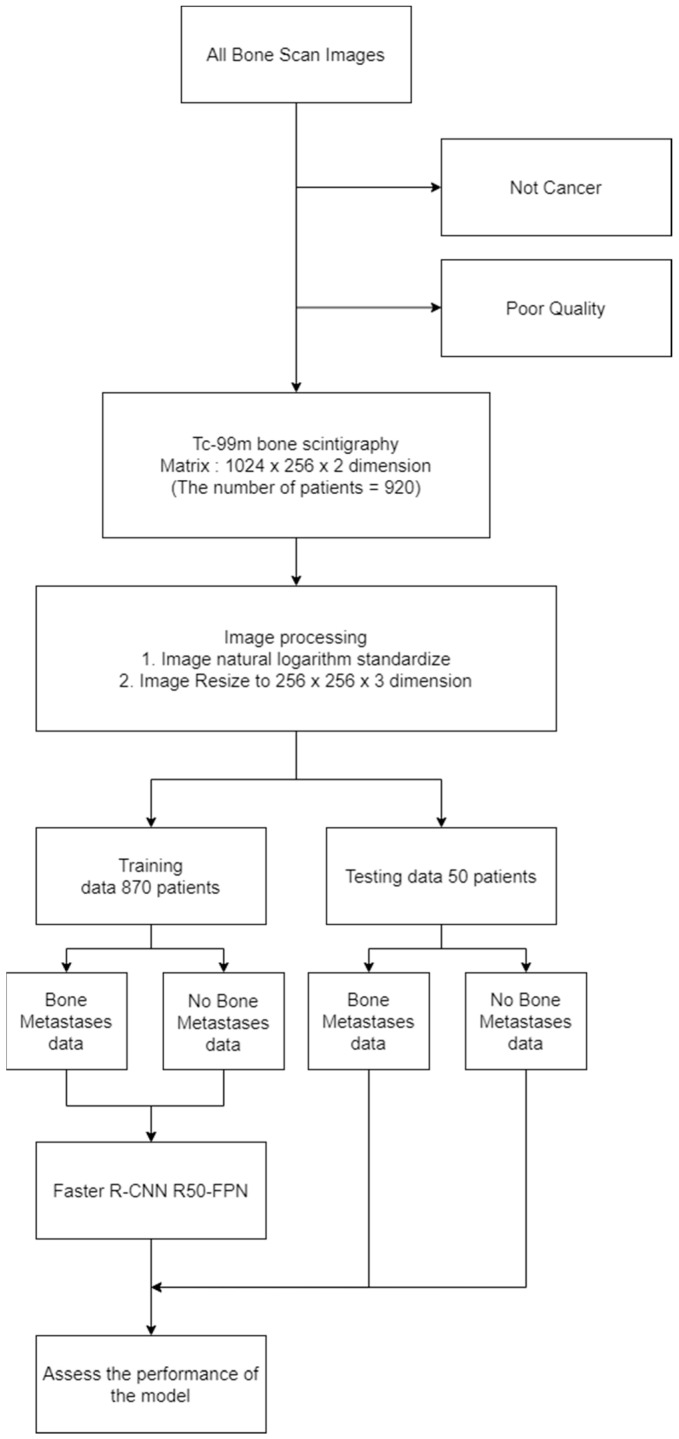
Flowchart for this study.

As presented in the flowchart in Figure 2, we directly process the original image without saving it in image formats such as portable network graphics (PNG) or joint photographic experts group (JPG). After splitting, the data are input into the object detection model for determining the model performance.

### 2.4. Faster R-CNN

R-CNN uses a selective search to locate 2000–3000 region proposals, compress the extracted region proposals into the same size, input them into a CNN that is only used for feature extraction, and input them into a support vector machine (SVM) for classification and into a linear regression for bounding box correction.

**Figure 3:**
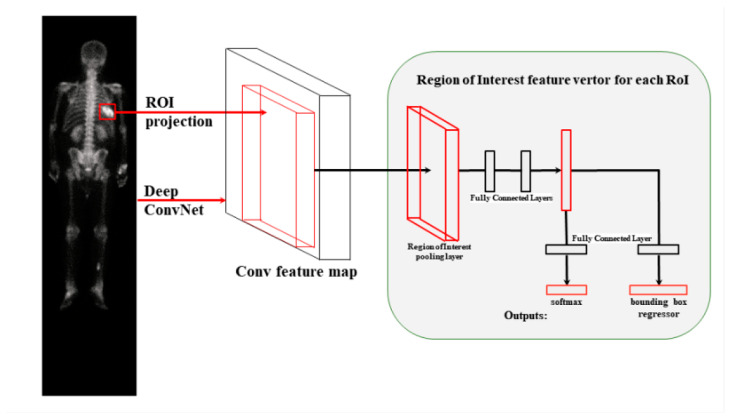
Faster R-CNN architecture.

Figure 3 presents the main architecture of Faster R-CNN. In this R-CNN model base, Fast R-CNN directly extracts features from the original input image and then uses RoIPooling for each region of interest (RoI) on the feature map of the entire image to extract a feature vector of a specific length. Fast R-CNN then removes the SVM and linear regression models, directly uses the fully connected layer for regression on feature vectors, and uses two fully connected layer branches to predict the class of RoI and its bounding box, thus improving the speed and prediction effect.

### 2.5. RPN

**Figure 4:**
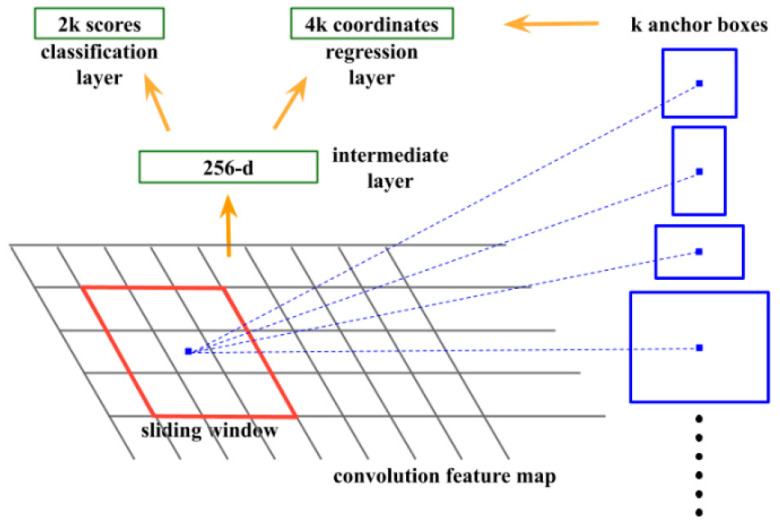
RPN architecture.

Figure 4 presents the main architecture of the RPN. An *n* × *n* sliding window operation on the feature map (see convolution feature map in Figure 4) generated by the backbone is performed, and the feature map within each sliding window is mapped to multiple proposal boxes (see the regression layer branch in Figure 4). Each box corresponds to the class information of whether an object is present or absent (see classification layer branch in Figure 4). RPN uses the natural sliding window operation of CNN as the feature extractor (see intermediate layer in Figure 4) and maps the feature map to a lower-dimensional feature map to save computation. To obtain the classes corresponding to boxes after the CNN operation, they are divided into two subnetworks. The input is the feature map output of the intermediate layer, which is a subnetwork responsible for box regression and for class regression. Since the features of each spatial position of the feature map generated by the intermediate layer are used to predict the presence of the class and the box of the class in the corresponding position of the window before mapping, a 1 × 1 CNN is used for calculation (the same as the fully connected layer), which is performed using RPN. All sliding window positions share an intermediate layer and subsequent branch networks for classification and box regression.

Since the sliding window operation is implemented using a square CNN convolution, to train the network to accept objects of different aspects, ratios, and sizes, RPN introduces the concept of anchor boxes. In each sliding window position, k anchor boxes are preset. The position of each anchor box is the center point of the sliding window, and the aspect ratio and size of the k anchor boxes differ. The classification branch and the bounding box regression branch map the tensor of each spatial position of the feature map output from the intermediate layer into k bounding boxes and the corresponding categories. Assuming that the number of anchor boxes at each position is k, the feature vector output by the classification branch is 2k, and the output of the bounding box regression branch is 4k (the bounding box information, the *x* coordinate of the bounding box center point, the *y* coordinate of the bounding box center point, the bounding box width w, and the bounding box height h). The position (x, y, w, h) predicted by the bounding box branch is offset relative to the anchor box. From a functional perspective, the role of the anchor box is somewhat similar to the role of the proposal box provided to Fast R-CNN, and it also represents the possible location bounding box of the target. However, the anchor box is uniformly sampled, and the proposal box is extracted by feature extraction (or including in training). Therefore, the anchor boxes and the predicted bounding boxes have a one-to-one correspondence. From the relationship between the anchor boxes and the intersection over union (IoU) of the ground truth bounding boxes, the positive and negative sample categories of each predicted bounding box can be determined. By assigning specific bounding boxes to objects in specific positions, sizes, and aspect ratios in a supervised manner, the model learns to fit objects of different sizes. Since the predicted bounding boxes are offset relative to the anchor boxes and the anchor boxes are evenly distributed on the feature map, only the predicted bounding box corresponding to the anchor box whose distance and size are close to the ground truth bounding box (larger IoU). The loss is calculated with the ground truth bounding box, which considerably simplifies the training. Otherwise, large numbers of predicted bounding boxes and ground truth bounding boxes are necessary to calculate the loss, especially in the initial stage of training.

### 2.6. Faster R-CNN ResNet50

On the Faster R-CNN base, the backbone is replaced by ResNet50. The feature maps are used by RPN and Fast R-CNN. The original multi-scale feature maps are from the C2, C3, C4, and C5 stages in ResNet (i.e., the outputs from the conv2_x, conv3_x, conv4_x, and conv5_x stages, respectively).

### 2.7. Faster R-CNN R50-FPN

**Figure 5:**
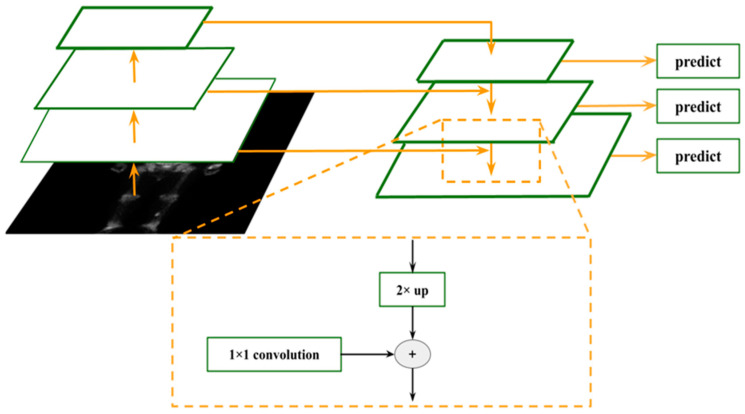
A building block illustrating the lateral connection and the top-down pathway merged by addition.

On the basis of Faster R-CNN ResNet, the FPN module is introduced, which uses the feature pyramid characteristics of CNN to simulate the image pyramid function, so that RPN and Fast R-CNN can be used in the multiple scale level feature map. Predicting objects of different sizes considerably improves the detection ability of Faster R-CNN. The inference time is saved compared with that of image pyramids. The principle is illustrated in Figure 5.

**Figure 6:**
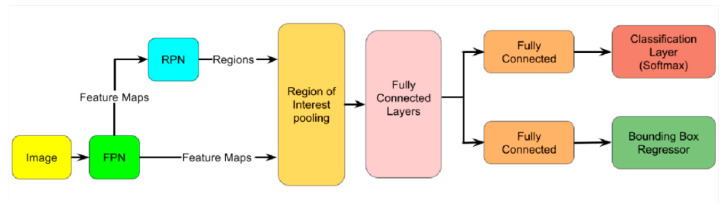
Faster R-CNN FPN architecture overview.

As shown in Figure 5, FPN does not simply use the feature maps output by multiple CNN layers of the backbone for bounding box regression and classification. Rather, it fuses the feature maps of different layers in the form of top-down and lateral connections for later use. Therefore, the deep semantic low-resolution features generated by CNN network forward propagation are fused with shallow semantic high-resolution features, thereby compensating for the lack of semantic abstraction of low-level features, which is similar to adding context information. Among the top-down process uses the nearest neighbor interpolation to upsample the low-resolution feature map to the same size as the lower-level feature map, with the fusion of the maps, and the lateral connection uses a resolution of 1 × 1 for the lower-level feature map. The convolution is scaled to the same number of channels as the upper layer feature map for their fusion, and pixel-level addition is then performed. The fused feature map is used for prediction and continues to propagate in the top-down direction for the feature fusion of lower layers until the last layer. Figure 6 presents the architecture of Faster R-CNN combined with R50-FPN.

### 2.8. Swin Transformer and YOLOR

Swin Transformer proposes a backbone that can be widely used in all computer vision fields. Most of the common hyper parameters in CNN networks can also be manually adjusted in the Swin Transformer, such as the number of network blocks, the number of layers per block, and the size of the input image. Both ViT and iGPT use images of small size as input, and this direct resize strategy leads to the loss of considerable information. By contrast, the input of the Swin Transformer is the original size of the image. The hierarchical structure of Swin Transformer also confers the ability to perform segmentation or detection tasks with structures, such as FPN.

YOLOR constitutes a completely different set of neural networks. It serves as a memory plug-in and can store all the information of the input model. When performing multiple tasks, the model can extract the required information without requiring training the model from scratch, as in traditional methods. Similar to the human brain, it combines consciously learned explicit knowledge and unintentionally absorbed implicit knowledge. When used to answer relevant questions, it can retrieve key information from tacit knowledge.

### 2.9. Model Estimation

Dice similarity coefficient (*DSC*) is mainly used as the evaluation standard of object detection. *DSC* (Equation (1)) is a set similarity measurement target, which is usually used to calculate the similarity of two samples. Values range from 0 to 1, with values of 1 denoting best and 0 denoting worst. In object detection, predictions can be classified as true positives (*TP*), true negatives (*TN*), false positives (*FP*), or false negatives (*FN*). In the case of bone metastasis detection, *TP* indicates that the predicted bounding box is correctly classified and has detected bone metastasis. *FP* denotes that the predicted bounding box has not detected bone metastases. *FN* indicates that a ground truth is present in the image and the model failed to detect the object. *TN* denotes every part of the image where we did not predict an object. This metric is not useful for object detection. Hence, we ignore *TN*.

(1) $$DSC\left(P,\;T\right)=\frac{2*TP}{\left(FP+2*TP+FN\right)}\;\;$$

**Figure 7:**
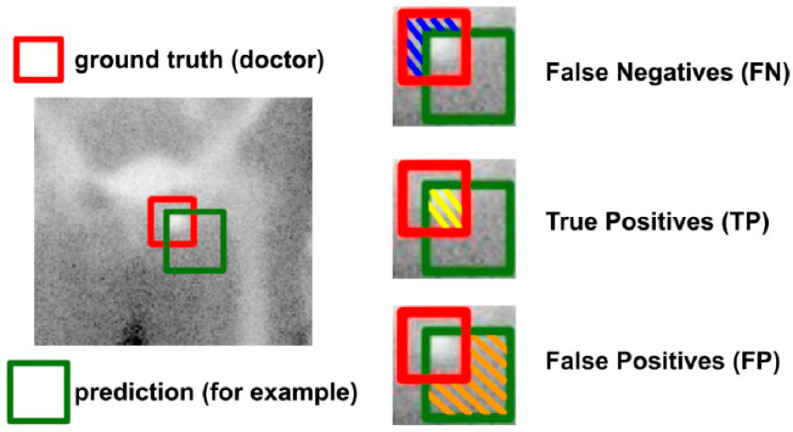
Dice Similarity Coefficient.

In Figure 7, the ground truth (doctor) is the red bounding box and the prediction is the green bounding box. The intersection of the two bounding boxes is *TP*. The bounding box of ground truth is detected, but the bounding box of the prediction is not detected, indicating *FN*. The bounding box of ground truth is not detected, but the bounding box of the prediction is detected, indicating *FP*. *TN* are beyond the scope of the two bounding boxes and are included in the calculation of *DSC*.

## 3. Results

### 3.1. Results of Object Detection Model and Doctor

We invited three senior nuclear medicine physicians (doctor A, doctor B, and doctor C. Their details are presented in Table 1) with more than 5 years of clinical experience to annotate 50 cases of test data. In a blinded manner, physicians annotated the front and back of patients’ bone scan images with a confidence level of more than 80%. The special emphasis here entails physicians without referring to patients’ clinical data, such as medical records, cancer types, tumor markers, PET/CT images, and MRI images. These ground truths and our object detection model predicted the DSC of 50 test data case results according to the different thresholds (0–1) of the AI model prediction confidence value (see Table 1).

In Table 1, we found that the doctor and AI performed best at a threshold of 0.7. Although our DSC performance is inadequate, we used the drawing result of one physician as the ground truth and the drawing result of another physician as the prediction result. Our highest DSC was 0.6640 (see Table 1), and the highest DSCs among the physicians was 0.7040 (see Table 2), denoting a difference of only 0.04 between the DSC values obtained using our model and the physicians’ value.

### 3.2. Results of Different Doctors

Calculations of the DSC results between two physicians are presented in Table 2.

**Figure 8:**
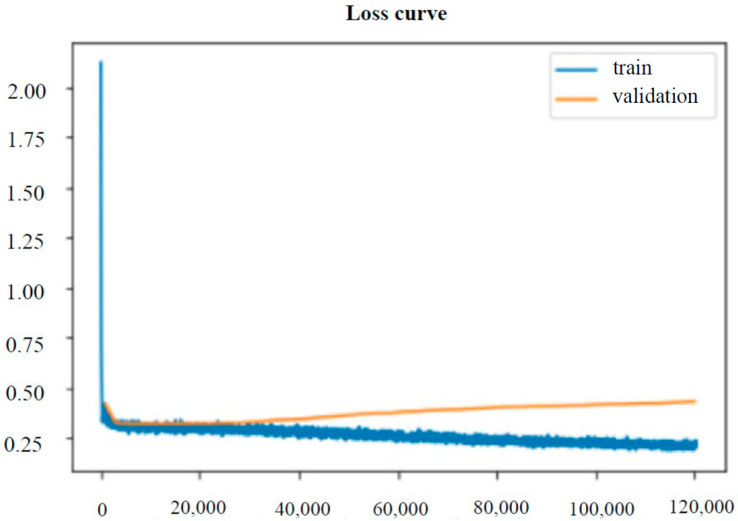
Training and validation loss form Faster R-CNN R50-FPN Model. Table 1. The results of Different threshold Faster R-CNN R50-FPN Model and Doctor.

Our Loss Function figure of Faster R-CNN R50-FPN Model is presented in Figure 8. The horizontal axis denotes iteration, and the vertical axis denotes loss. From Figure 8, it can be observed that the model has successfully learned the features, and that the loss has converged. Since our loss has dropped significantly, and we have not seen a rise in loss. Therefore, I have enough confidence to judge that the AI model has successfully learned.

### 3.3. Results of Different Object Detection Models

We investigated the models Faster-RCNN X101-32x8d-FPN, Faster R-CNN Swin-T FPN, YOLOR, and Faster R-CNN RPN R50-FPN and choose the highest DSC of threshold for each model. The highest DSC was obtained for Faster R-CNN R50-FPN (see Table 3). The DSC of Faster R-CNN Swin-T FPN and YOLOR were very similar and very poor because neither model DSC all performed poorly. We can see from the performance of DSC that the model has not successfully learned effective features.

## 4. Discussion

The results indicate that our object detection model had the highest DSC was 0.6640, and the highest DSC among the physicians was 0.7040, denoting a difference of only 0.04 between the DSC values obtained using our model and physicians. Since the gap between our model results and physicians is minuscule, it is close to the standard of physicians. In experimental results, the doctors supported this result. Senior physicians could clearly identify bone metastases, and some physicians labeled more bounding boxes to prevent undetected metastasis cases.

Computer-aided systems that automatically detect the location of metastatic lesions on bone scans are uncommon. The most well-known commercial software is the Bone Scan Index (BSI) [15]. BSI, developed using an artificial neural network, detects bone metastases in patients with prostate cancer through image segmentation, identifies areas of bone with increased radiopharmaceutical uptake, and classifies these areas as having malignant or benign lesions. Hsieh et al. used deep learning to classify bone metastases in bone scans [16]. Liu et al. used deep learning to segment bone metastases on bone scans [17]. Zhang et al. used improved U-NET algorithms to achieve bone metastasis segmentation [18]. Cheng et al. detected bone metastases using object detection in the chest bone scan images of prostate cancer [19]. Few studies using object detection techniques have been conducted.

Our study has some limitations. First, we could not focus on every single cancer, because the data are not enough. The dataset was only collected from patients in a tertiary academic medical center because manual labeling is time-consuming and requires physicians in multiple medical centers. Second, the size and numbers of bounding boxes annotated by each physician varied. The area was calculated according to the formula of DSC (see Figure 7), which substantially affects the DSC of the model. Third, in our research, annotation was performed in a blinded manner without reference to patient clinical data, such as reports, cancer types, tumor markers, CT images, and MRI images. If physicians could refer to this information, the DSC between physicians may have been improved. The same applies for the model. Sex and cancer type have a substantial impact on the results. For example, the results of male prostate cancer and female breast cancer vary considerably. Sensitivity is the highest for prostate cancer, which is mainly osteoblastic. The detection of breast and lung cancer is also notably high, although these tumors exhibit relatively mixed lesion patterns. Future models that incorporate this information may exhibit improved accuracy. Fourth, the model does simultaneously analyze the relationship between the front and back sides of bone scan images. When the front and back sides of bone scan images are different, they are analyzed by the training and testing models separately. However, physicians can interpret the front and back sides of bone scan images simultaneously, and this results in annotation differences. This affects the number of bounding boxes and results in a decrease in DSC. Furthermore, only a minority of patients had bone lesions that could be differentiated by other advanced medical imaging such as CT, PET/CT, or MRI.

The AI we developed does not need additional clinical information and has an interpretation accuracy comparable to that of nuclear medicine physicians without access to extra information. This makes it a useful tool for initial screening of bone metastases and can support clinical work, while reducing barriers for non-nuclear medicine physician researchers in organizing research data. However, it cannot replace the conventional interpretation carried out by nuclear medicine physicians who have access to additional clinical information.

## 5. Conclusions

Our research is the first to use object detection on the whole body bone scan image to detect the metastases site from cancers. Demonstrated that object detection technology can assist in the safe detection of bone metastases. The results of the object detection techniques were similar to the results of physicians, and object detection technology can thus reduce the number of undetected lesions caused by physician fatigue. Object detection can also assist oncologist in the annotation of lesions for treatment.

## Figures and Tables

**Table 1:** The results of Different threshold Faster R-CNN R50-FPN Model and Doctor.

Doctor	Threshold	DSC	Doctor	Threshold	DSC	Doctor	Threshold	DSC
doctor A	0	0.0861	doctor B	0	0.0771	doctor C	0	0.1532
doctor A	0.1	0.3009	doctor B	0.1	0.2970	doctor C	0.1	0.3601
doctor A	0.2	0.4303	doctor B	0.2	0.4337	doctor C	0.2	0.4519
doctor A	0.3	0.5174	doctor B	0.3	0.5299	doctor C	0.3	0.5318
doctor A	0.4	0.5819	doctor B	0.4	0.5915	doctor C	0.4	0.5885
doctor A	0.5	0.5784	doctor B	0.5	0.5924	doctor C	0.5	0.5707
doctor A	0.6	0.6021	doctor B	0.6	0.6098	doctor C	0.6	0.5711
doctor A	0.7	0.6640	doctor B	0.7	0.6210	doctor C	0.7	0.5750
doctor A	0.8	0.6445	doctor B	0.8	0.6103	doctor C	0.8	0.5515
doctor A	0.9	0.5890	doctor B	0.9	0.5342	doctor C	0.9	0.4656
doctor A	1	0.4800	doctor B	1	0.4600	doctor C	1	0.3800

**Table 2:** The results of Different doctors.

Doctor Be Prediction	Doctor Be Ground Truth	DSC
doctor A	doctor B	0.7040
doctor B	doctor C	0.6545
doctor C	doctor A	0.6822

**Table 3:** DSC Results of Different Object Detection Model.

	Faster R-CNN R50-FPN	Faster-RCNN X101-32x8d-FPN	Faster R-CNN Swin-T FPN	YOLOR
doctor A	0.6640	0.5327	0.38	0.3827
doctor B	0.6210	0.6072	0.48	0.4832
doctor C	0.5750	0.5892	0.46	0.4622

## Data Availability

Not applicable.
